# Recent Advances in Spectroscopy and Imaging Techniques for Nondestructive Detection of Meat Quality and Safety

**DOI:** 10.1002/fsn3.71303

**Published:** 2025-12-06

**Authors:** Yushan Jiang, Dachen Wang, Zijie Wang, Huang Dai, Chen Wang, Yingli Wang

**Affiliations:** ^1^ College of Engineering Huazhong Agricultural University Wuhan China; ^2^ Co‐Innovation Center of Efficient Processing and Utilization of Forest Resources Nanjing Forestry University Nanjing China; ^3^ Key Laboratory of on Site Processing Equipment for Agricultural Products, Ministry of Agriculture, College of Biosystems Engineering and Food Science Zhejiang University Hangzhou China; ^4^ College of Mechanical and Electronic Engineering Nanjing Forestry University Nanjing China; ^5^ College of Food Science and Engineering Wuhan Polytechnic University Wuhan China; ^6^ School of Food and Biological Engineering Jiangsu University Zhenjiang China

**Keywords:** data analysis methods, imaging inspection, meat quality and safety, spectroscopic inspection

## Abstract

Meat is one of the most important foods in the human diet and is a major source of animal protein. It is essential to detect meat quality and safety for ensuring that high‐quality foods are delivered to consumers. Advanced spectroscopic techniques (near‐infrared spectroscopy, Raman spectroscopy, fluorescence spectroscopy, and terahertz spectroscopy) and imaging techniques (hyperspectral imaging, multispectral imaging, X‐ray imaging, and thermal imaging) provide feasible means for testing organoleptic properties, chemical composition, physicochemical properties, and safety indicators of meat. This review aimed to summarize the latest developments of spectroscopic and imaging techniques for meat quality and safety detection. The principles, multi‐scenario applications, advantages, disadvantages, and future prospects of these techniques are discussed. Spectroscopic techniques have been demonstrated to accurately detect changes in the chemical constituents and physical properties of meat, but can only perceive localized sample information. Imaging techniques provide visual information on the spatial distribution as well as physical and chemical characteristics of meat, but have a slower detection rate. Despite the remarkable outcomes attained by spectroscopy and imaging techniques in laboratory settings, their industrial applications remain encumbered by challenges, including substantial expenses and intricate data analysis procedures. In order to improve the comprehensiveness and accuracy of detection, future research directions might focus on the integration of multiple techniques combined with deep learning algorithms.

## Graphical abstract

A summary of the advanced techniques for meat quality and safety detection.

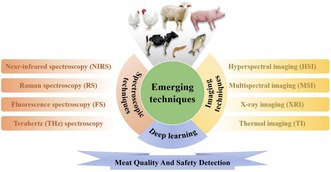

## 1 Introduction

Meat constitutes a pivotal component of the human diet, serving as a primary source of high‐quality proteins, vitamins, minerals, and other essential nutrients that promote human health (Kutsanedzie et al. 2019; Li et al. 2016; Xiong et al. 2017). In recent years, socio‐economic advancements and improved living standards have shifted consumer concerns toward the quality and safety of meat products. The procurement of reliable information regarding meat quality and safety is instrumental in the formulation of rational processing strategies and the optimization of production processes and storage conditions. Therefore, the quality and safety of meat are ensured (Wu et al. 2022; Zhai et al. 2019). The quality of meat is generally the synthesis of physical and chemical characteristics related to the appearance, palatability, and nutritional value (Huang et al. 2016). The safety of meat is to ensure that meat does not contain harmful substances or pathogens and that it does not cause acute or chronic harm to consumer health. Common parameters for evaluating meat quality and safety include moisture content, fat content, pH, protein content, and microbial load (Peng and Dhakal 2015). In recent years, sensory evaluation, chemical analysis, and microbiological methods have been developed for the objective measurement of meat quality and safety characteristics (Li, Sun, et al. 2021; Wang et al. 2018; Zhai et al. 2019). Sensory evaluation is time‐consuming and subjective, making consensus among evaluators difficult (Runu et al. 2016). Furthermore, sensors utilized in electronic noses and tongues depend on sophisticated data processing algorithms (Sipos et al. 2020), which may not adequately detect all target compounds (Vanaraj et al. 2025). Chemical analysis is susceptible to contamination, the complexity of the operational requirements, the hazards of the reagents, and the cost (Li et al. 2023). Microbial detection methods require extensive sample pretreatment, high consumption of chemical reagents, long detection times, and high costs (Pophiwa et al. 2020). In order to comply with the demands of the contemporary meat industry, it is imperative to formulate nondestructive, precise, and expeditious techniques for evaluating meat quality and safety.

Compared with traditional techniques, spectroscopy and imaging techniques allow for real‐time assessment of key meat quality and safety indicators during the production process, while avoiding sample damage and significantly improving testing efficiency and accuracy (Wang et al. 2018). According to wavelength, the electromagnetic spectrum is classified as γ‐rays, X‐rays, ultraviolet, visible light, infrared, microwave, and radio waves (Zhang, Yang, et al. 2024; Zhang, Luan, et al. 2024). Reflection, absorption, transmission, and photoluminescence occur when light of different wavelengths is incident on meat. Near‐infrared spectroscopy (NIRS), Raman spectroscopy (RS), fluorescence spectroscopy (FS), and terahertz (THz) spectroscopy based on different optical phenomena hold great promise in quality evaluation, chemical analysis, grading, and safety and freshness determination (Prieto et al. 2017). Unlike one‐dimensional spectral data, imaging technology can obtain spectral‐spatial information related to the physical, chemical, and sensory properties of meat products (Wu et al. 2022; Xiong et al. 2017). Emerging hyperspectral imaging (HSI), multispectral imaging (MSI), X‐ray imaging (XRI), and thermal imaging (TI) are often used to assess meat quality and safety. Based on the acquired spectral and image data, multiple quality parameter prediction models are built in combination with chemometrics or deep learning (DL) algorithms (Aheto et al. 2020, 2019). The coefficient of determination ($R^{2}$), correlation of coefficient (*r*), and root‐mean‐square error (RMSE) are commonly utilized evaluation metrics of model performance.

This paper aims to methodically synthesize the spectroscopic techniques (NIRS, RS, FS, and terahertz spectroscopy) and imaging techniques (HSI, MSI, XRI, and thermal imaging) commonly used in recent years for assessing product quality and safety. A comprehensive overview is provided of these emerging technologies for evaluating the quality and safety of various meats, including pork, beef, lamb, chicken, fish, and shrimp. The theoretical foundations, current applications, distinctive features, and challenges related to the industrial implementation of these technologies are thoroughly examined. The benefits and limitations of integrating spectroscopic and imaging techniques with DL algorithms for detecting meat quality and safety are analyzed, and future research directions are outlined.

## 2 Spectroscopic Techniques

### 2.1 Near‐Infrared Spectroscopy

**FIGURE 1:**
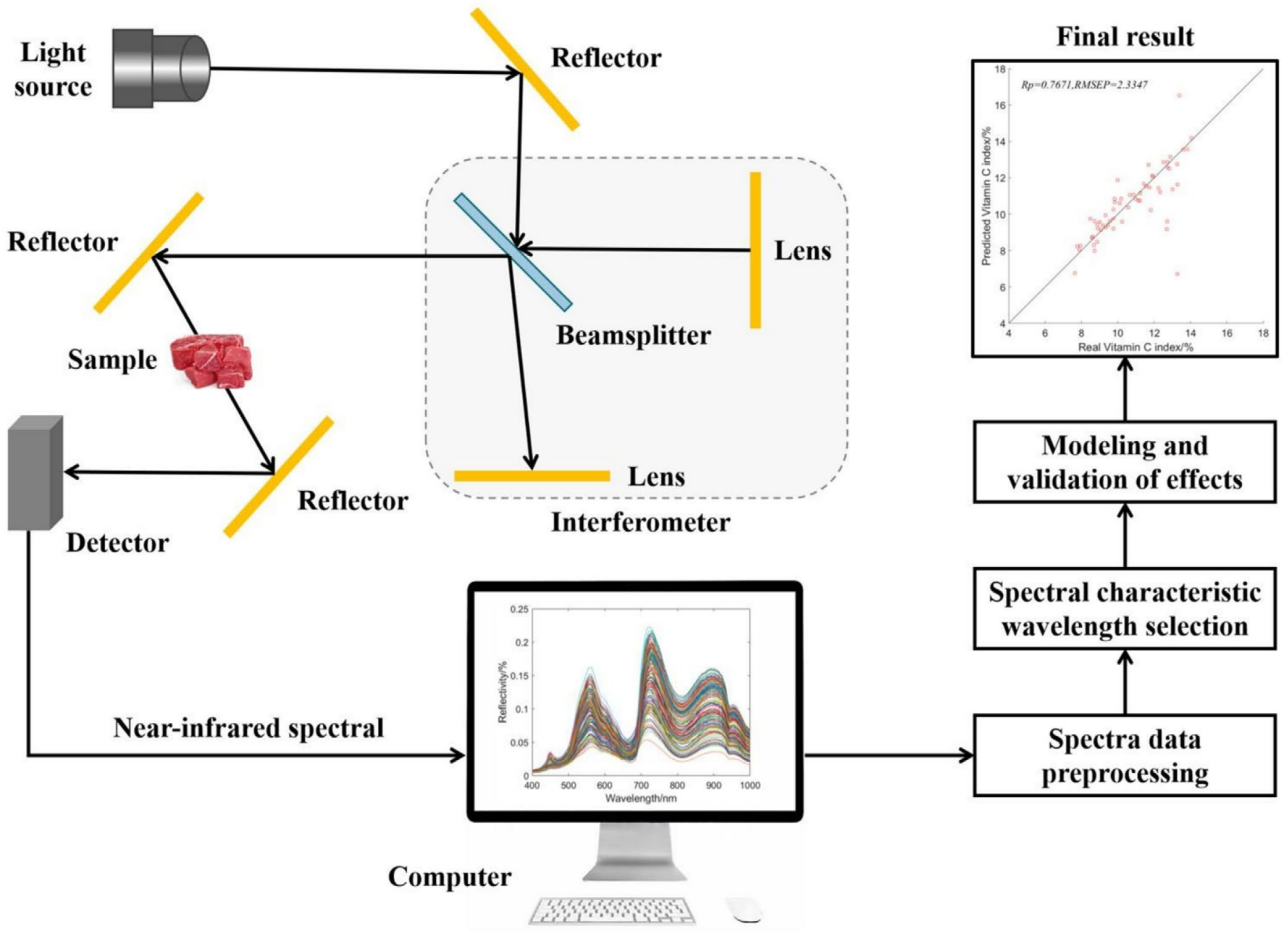
Basic components of a near‐infrared spectral acquisition system.

The near‐infrared (NIR) region represents an electromagnetic wave situated between the visible and mid‐infrared spectra, with wavelengths ranging from 800 to 2500 nm (Wang, Xu, et al. 2022). Different organic compounds typically contain distinct hydrogen‐containing groups, such as O–H bonds in water, N–H bonds in proteins, and C–H bonds in fats. Upon exposure to the near‐infrared light, these groups are excited into vibrational resonance, leading to energy absorption (Zareef, Arslan, Hassan, Mehedi Hassan, et al. 2021). Each molecule absorbs energy at specific wavelengths at varying rates, resulting in characteristic peaks and valleys within the spectrum. Components are then analyzed qualitatively or quantitatively based on the position and intensity of the spectra. As shown in Figure 1, a typical NIR spectrometer consists of a light source, a beam splitting system, a detector, and a data processing system. Figure 1 also shows the workflow of NIR spectroscopy.

NIRS is capable of providing extensive information regarding meat quality and safety (Li, Wu, et al. 2022; Xu et al. 2018). It represents a rapid, nondestructive method for assessing meat (Xie et al. 2015). NIRS is frequently combined with multivariate algorithms to extract chemical information, preprocess spectra to remove redundant data, and model the characteristics of meat quality and safety (Pan et al. 2015). Model performance is typically evaluated using metrics such as the coefficients of determination for the prediction set ($R_{p}^{2}$) and the calibration set ($R_{c}^{2}$), the root‐mean‐square error of the prediction (RMSEP), and the root‐mean‐square error of cross‐validation (RMSECV) (Kutsanedzie et al. 2018; Li et al. 2015). Higher $R_{p}^{2}$ and $R_{c}^{2}$ values, along with lower RMSEP and RMSECV, indicate superior predictive performance of the model for the target meat quality and safety variables (Chen et al. 2015). Zuo et al. (2023) utilized NIR HSI to non‐invasively detect the nutrient content and its distribution in pork. The results demonstrated that the key wavelength variables extracted using the standard normal variable transform combined with the competitive adaptive reweighted sampling algorithm exhibited superior predictive performance. To enable non‐invasive detection of meat quality and safety, it is essential to integrate various algorithms with the NIRS system. However, since the predictive performance of attribute models depends on the specific algorithm integrated with the NIRS system (Szymańska et al. 2015), it is necessary to experiment with different algorithms and select the one with the best performance.

The NIR system, combined with advanced data processing algorithms, is employed to evaluate critical physical characteristics of meat, including pH and Warner–Bratzler (WB) shear force (Ouyang et al. 2022; Wei et al. 2022). Among the most commonly employed data processing techniques are principal component analysis (PCA), linear discriminant analysis (LDA), partial least‐squares regression (PLSR), and support vector machine (SVM). Feng et al. (2024) monitored the TBARS of ground beef based on the data fusion of NIRS and paper chromogenic array. A standardized paper chromogenic array was fabricated by photolithography with nine chemically chromogenic dyes to capture changes in volatile organic compounds (VOCs) in ground beef during storage. PLSR demonstrated the best performance in the optimized model, achieving a *R* ^2^ of 0.9477. This suggests that the combination of NIR and PCA has the potential to monitor TBARS values to assess the freshness of ground beef.

NIRS analysis integrates multiple advanced technologies, such as spectral measurement, computer science, chemometrics, and fundamental testing methodologies. Through the judicious selection of chemometric techniques, NIRS establishes a correlation between spectral data and reference index value. This process results in a highly accurate and stable mathematical model capable of predicting the reference values of unknown samples. Notably, this method offers significant advantages, including nondestructiveness, convenience, rapidity, and online detection capabilities (Guo, Barimah, et al. 2020; Guo, Wang, et al. 2020). In recent years, NIRS has gained prominence in the field of rapid nondestructive testing for meat quality and safety. Currently, NIRS‐based nutrient content detection has reached a relatively mature state, exhibiting high precision in meat grading, identification, and index detection. Despite its rapid advancement, NIRS technology still encounters several limitations. The accuracy of the models depends heavily on time‐consuming and labor‐intensive calibration processes. Moreover, updating these models requires substantial investments of time, effort, and financial resources. Additionally, in industrial applications, factors such as light scattering in solids or opaque liquids, variations in temperature, density, and particle size, as well as optical noise, can alter the NIR spectrum, leading to baseline shifts (Wang et al. 2018; Wu et al. 2022).

### 2.2 Raman Spectroscopy

**FIGURE 2:**
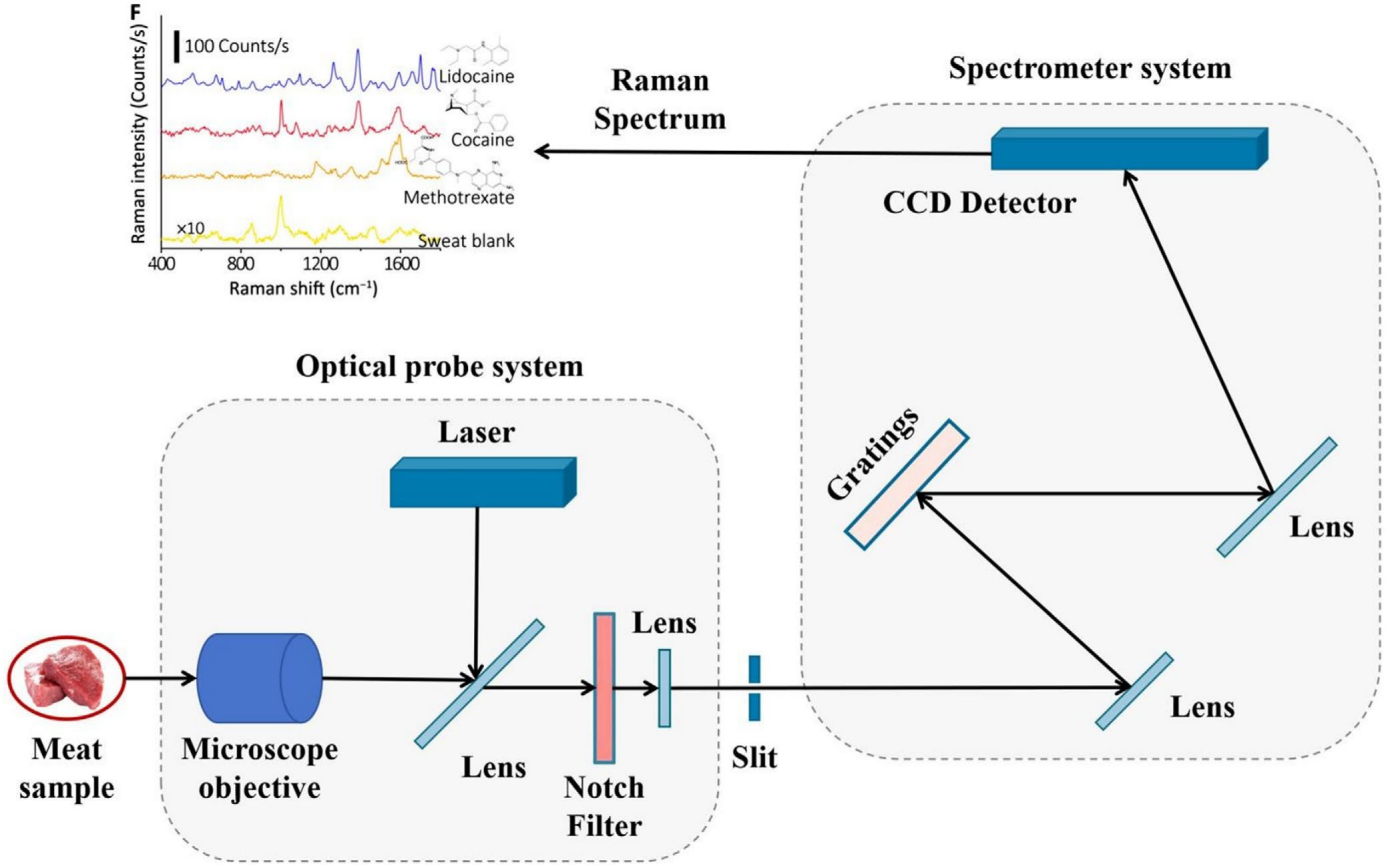
Basic components and principles of Raman spectroscopy.

RS techniques employ lasers to probe molecular vibrational and rotational modes (Wang, Chen, et al. 2021). The fundamental principle posits that when a monochromatic laser beam is directed at a specific sample molecule, the photons interact with the molecule's intrinsic vibrational, rotational, or other low‐frequency modes. Most incident photons (99.9999%) scatter at the same frequency as the incident light, a phenomenon referred to as Rayleigh scattering or elastic scattering. In rare cases (0.0001%), a small fraction of the light changes frequency, resulting in Raman scattering (Ember et al. 2017; Yaseen et al. 2017). Distinct chemical compositions yield unique peaks in Raman spectra (Qiu et al. 2024). A typical Raman spectrometer configuration includes a laser source, a detection system, a dispersion system, and an optical system, as shown in Figure 2.

Recently, the application of RS in the assessment of meat quality and safety has significantly increased (Nunekpeku et al. 2025; Wang, Chen, et al. 2021; Zhai et al. 2019). Iqbal et al. (2025) prepared different batches of pure beef meatballs and meatballs with varying degrees of adulteration. These samples were then scanned by RS in both intact and cut form. These samples were then subjected to a thorough analysis using a PLS‐DA model, which yielded accuracy levels ranging from 52.50% to 85.00%. Bai et al. (2022) employed Raman scattering and chemometrics to predict lipid degradation and storage time of chilled beef flanks. The results demonstrated a close relationship between lipid degradation and the storage time of chilled meat products. Consequently, RS has been shown to possess the capability to predict the storage duration of chilled beef flanks. In recent years, RS has seen a surge in applications for the prediction of meat quality and safety, with notable advancements in accuracy.

RS for meat grading and identification faces the same challenge of noisy spectral data as NIRS. The application of chemometric methods to preprocess Raman spectra effectively reduces spectral noise, thereby enhancing the accuracy of predictive models (Jiao et al. 2021; Zhu et al. 2018). Berhe et al. (2015) employed a combination of Raman scattering spectroscopy and multivariate data analysis techniques, such as PCA and PLS‐DA, to explore the relationship between heating temperature and duration, as well as storage periods during the processing and production of cooked meat. Their findings indicated that RS achieved an accuracy of 97.87% in predicting the end‐point temperatures (EPTs) of heat‐treated meat products. Robert, Fraser‐Miller, et al. (2021) employed RS in conjunction with three chemometric techniques to differentiate between beef, lamb, and venison samples. PLS‐DA and SVM classification were utilized in building classification models for meat discrimination, whereas PCA was used for exploratory studies. The PLS‐DA model yielded more than 80% accuracy in classifying each type of meat. This suggests that the combination of chemometric methods may enhance the precision of RS in assessing meat quality and safety.

Currently, RS has been increasingly employed in meat research. The stability and accuracy of prediction models established through the integration of various chemometric techniques are superior. Nevertheless, considering the intrinsic heterogeneity and complexity of meat and meat products, the development of comprehensive databases and their correlation with diverse indicators remains a critical requirement for future investigations.

### 2.3 Fluorescence Spectroscopy

**FIGURE 3:**
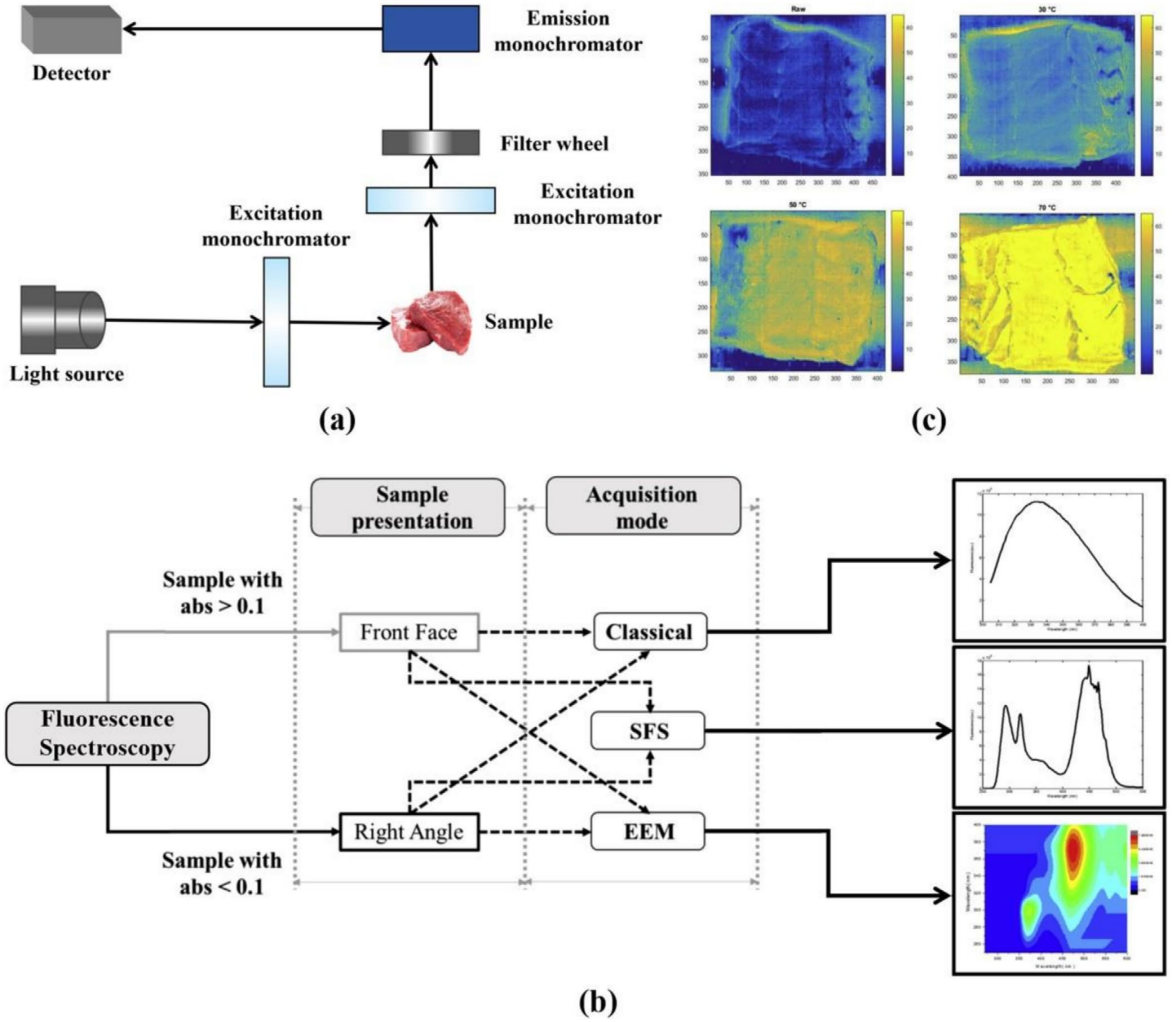
(a) The basic components of a fluorescence spectrometer. (b) Primary acquisition modes for FS. (c) FS imaging of a cod sample heated at different temperatures (Hassoun et al. 2019).

FS is a non‐invasive technique utilized to determine the fluorescent properties of objects (Gu et al. 2022; Lenhardt et al. 2015). When a fluorophore in food is exposed to light at a specific wavelength, commonly referred to as excitation light, the electrons within the molecule transition from their ground state to a higher excited state. Subsequently, these excited electrons release energy in the form of emitted light as they return to the ground state. The emitted light during this process is fluorescence, and the molecules that exhibit this phenomenon are known as fluorophores (Momin et al. 2023). FS can provide detailed information on fluorophores present in both simple and complex biological matrices (Ahmad et al. 2016). Certain lipid oxidation products in meat possess fluorescent properties, including protoporphyrin IX in collagen, tryptophan residues in proteins, and vitamin A in meat fat (Hassoun et al. 2019; Sahar et al. 2016). A fluorescence spectrometer generally consists of an excitation light source, a detector, a sample chamber, a grating, and a data processing system, as shown in Figure 3a. The acquisition mode is presented in Figure 3b.

The data acquired through FS are typically presented in the form of an emission spectrum. This spectrum is generated by measuring the emitted light (fluorescence) across a broad wavelength range under excitation at a fixed wavelength, followed by plotting the fluorescence intensity against the emission wavelengths (in units of nm). In food analysis, frontal fluorescence spectroscopy (FFS) represents a promising technique (Radotić et al. 2023). In FFS, only the surface of the sample is illuminated with excitation light and excited fluorescence is measured. This approach minimizes interference from reflected light, scattered radiation, and depolarization effects (Peters and Rapp 2024).

FS combined with chemometrics has extensive applications in various aspects of meat product analysis, including identification (Saleem et al. 2024), categorization (Karoui et al. 2017), storage (Sahar et al. 2016), and processing (Hassoun and Karoui 2015). Boughattas et al. (2019) applied FFS alongside chemometric tools such as PCA and FDA to detect pure canned tuna and its adulterated mixtures at varying percentages. Their study demonstrated the ability of this technique to distinguish between skipjack, yellowfin tuna, and bigeye tuna, which are among the most commonly used species in canned tuna products. This approach holds promise as an online screening tool for canned authenticity verification. Hassoun et al. (2019) employed FS coupled with PLSR to monitor the cooking temperature of cod fillets at three temperatures (30°C, 50°C, and 70°C). The resulting spectra were visualized using a linear color scale, ranging from blue (representing low‐temperature samples) to yellow (representing high‐temperature samples). This research highlighted the potential of FS imaging for categorizing and visualizing the thermal state of fish. As shown in Figure 3c.

FS, a rapid and non‐invasive technique, exhibits considerable potential for practical applications in the future evaluation of meat quality and safety. Furthermore, this technique facilitates acquiring significant technical information concerning products stored under various preservation conditions. However, it is important to recognize the inherent limitations and challenges associated with FS. Specifically, not all materials possess intrinsic fluorophores capable of undergoing fluorescent excitation. Additionally, multiple fluorophores within a sample may result in spectral peak overlap, which can influence the accuracy of the results (Zou et al. 2016). To achieve more accurate and detailed chemical imaging and spectral data, it is imperative to allocate increased resources toward research and development efforts in this field.

### 2.4 Terahertz Spectroscopy

**FIGURE 4:**
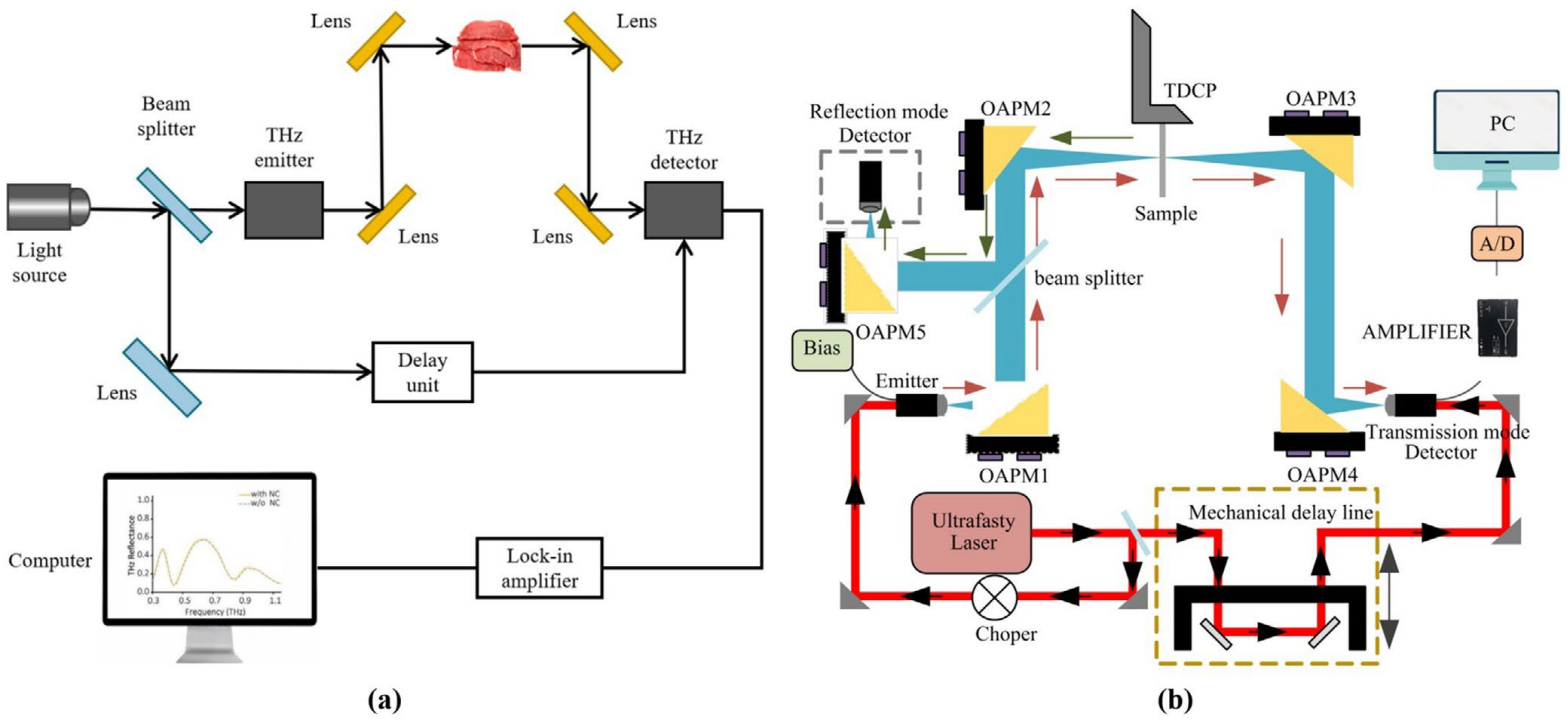
(a) Schematic diagram of a typical experimental THz spectroscopy setup. (b) Typical setup and schematic of a THz‐TDS system (Chu et al. 2023).

THz spectroscopy has emerged as a key development in spectroscopy technology, due to its unique radiation band. THz waves are classified as far‐infrared waves, with a frequency range spanning from 0.1 to 10 THz and corresponding wavelengths ranging from 0.03 to 3 mm. This frequency range is positioned between the millimeter‐wave and infrared spectra (Jang et al. 2024; Liu 2024). The unique properties of THz waves include fingerprint spectroscopic capabilities (Ren et al. 2020), low photon energy (Han et al. 2024), good penetration ability (Sun et al. 2021), and coherence (Afsah‐Hejri et al. 2020). A typical THz spectroscopy setup is shown in Figure 4a. The terahertz time‐domain spectroscopy (THz‐TDS) system comprises an ultrafast pulsed laser THz transmitter, a THz receiver, and a time‐delay control system (Wang, Xie, and Ying 2022), as shown in Figure 4b. Owing to their longer wavelengths compared to visible and near‐infrared (NIR) spectral bands, THz waves demonstrate reduced scattering tendencies. Additionally, THz waves exhibit significantly lower photon energy than X‐rays, making them non‐ionizing and safe for biological molecules, which renders them highly suitable for food detection applications (Tong et al. 2021).

The unique properties of THz waves allow them to probe molecular motions in the THz range that are characteristic of many chemical compounds and may not be as accessible in other spectral bands. These molecular dynamics play a crucial role in determining the quality of meat products. The difference in water content between muscle and fat tissue results in varying absorption rates of THz waves (Qi et al. 2019). Xiong et al. (2022) demonstrated that lean meat absorbs THz radiation, while fat meat is nearly transparent to it, attributable to the low‐energy responses of macromolecules, cells, and tissues. This property can be employed for quantifying the fat‐to‐lean ratio in meat products.

Presently, THz spectroscopy is undergoing substantial progress in the trace detection of harmful substances and their analogs in food. Wang et al. (2018) integrated THz‐TDS with PLSR to quantify the concentrations of chlortetracycline hydrochloride (CCH) and tetracycline hydrochloride (TCH) in chicken meat, achieving correlation coefficients exceeding 0.9. Wang et al. (2019) successfully localized metal contaminants in sausages using THz spectroscopy with PCA. Despite its promising potential, several challenges remain in the application of THz spectroscopy for meat quality and safety inspection.

Theoretical research in this domain is still in its nascent stages, and the experimental platforms for transmitting and receiving THz waves require further enhancement. In the future, the technology will need to undergo continuous optimization to adapt to the complex and evolving practical inspection environment. Table 1 summarizes the broad applications of spectroscopic techniques in meat quality and safety detection over the past few years.

**TABLE 1:** Summaries of different spectroscopic techniques applied for meat quality and safety detection.

Techniques	Samples	Testing parameters	Measured attributes	Chemometric procedures	Performance	References
Near‐infrared spectroscopy	Pork	400–700 nm	Metmyoglobin content	MSC, RF, and SPA	$R_{p}^{2}$ = 0.901, RMSEP = 0.0145, RMSE = 0.0216	Wang, Cai, et al. (2024)
	Beef	400–2500 nm	Classification	PCA and PLS‐DA	Accuracy of 100%	Liu, Xiang, et al. (2024)
	Beef	400–1000 nm	EAA	LSTM	$R_{p}^{2}$ = 0.9095, RPD = 2.76	Dong et al. (2024)
	Goat	1000–2500 nm	Characteristics of performance	SVMR	Accuracy of 96.7%, $R_{p}^{2}$ > 0.99	Vasconcelos et al. (2024)
	Lamb	680–2600 nm	Freshness	MSC, SNV, and PCA	Accuracy of 97.6%	Li, Wei, and Liang (2024)
	Freeze‐dried beef and mutton	1176–2703 nm	Intramuscular fat and protein	PCA and PLSR	$R_{p}^{2}$ = 0.984 and 0.965, RMSE = 0.74 and 0.76, respectively	Bailes et al. (2022)
	Chicken	900–1700 nm	Minced chicken gel strength	GA	$R_{c}^{2}$ = 0.9223, $R_{p}^{2}$ = 0.8772	Li, Nunekpeku, et al. (2025)
	Chicken	600–1000 nm	UMP	PLSR and MVN	${R_{p}}^{2}=$ 0.82, RMSE = 0.01	Kim et al. (2025)
Raman spectroscopy	Pork	1600–1700 cm^−1^	WHC and gel strength	UVE‐SVM	$R_{c}^{2}$ = 0.9047 and 0.9393, RMSEC = 1.1770 and 101.0498, $R_{p}^{2}$ = 0.8553 and 0.8508, RMSEP = 1.5468 and 164.8441, RPD = 2.1567 and 2.1981, respectively	Li, Zhang, et al. (2025)
	Pork	200–2000 cm^−1^	deoxymyoglobin and oxymyoglobin	RF‐PLS	*R*_*p*_ = 0.8936 and 0.9762, RMSEP = 2.91 and 1.23, RPD = 1.97 and 4.47, respectively	Li, Haruna, et al. (2022)
	Bull beef	1300–2800 cm^−1^	Sensory characteristics	PLSR	$R_{\mathrm{cv}}^{2}$ = 0.50–0.84, RMSECV = 1.31–9.07	Zhao et al. (2018)
	Beef	555–1800 cm^−1^	Adulteration	PLS‐DA	Accuracy of 52.50%–85.00%	Iqbal et al. (2025)
	Beef	750–1750 cm^−1^	pH and IMF	PLSR	$R_{c}^{2}$ = 0.79 and 0.75, RMSEC = 0.14 and 0.22, $R_{p}^{2}$ = 0.6 and 0.6, RMSEP = 0.27 and 0.3, $R_{\mathrm{cv}}^{2}$ = 0.58 and 0.72, RMSECV = 0.19 and 0.26, respectively	Robert, Jessep, et al. (2021)
	Chicken	400–3200 cm^−1^	Gel strength	LSTM	$R_{p}^{2}$ = 0.9882, RPD = 9.2091	Nunekpeku et al. (2025)
	Beef, lamb, and venison	313–1895 cm^−1^	Classification of species	PCA, SVM, and PLS‐DA	Accuracy of 80%–100% (validation)	Robert, Fraser‐Miller, et al. (2021)
	Beef, pork, and mutton	0–2000 cm^−1^	Identification of species	RF and BPNN	Accuracy of 99.42%	Sun et al. (2022)
Fluorescence spectroscopy	Pork	400–1000 nm	TVB‐N and pH	PLSR	Same temperature (−24°C): $R_{p}^{2}$ = 0.9487 and 0.9693, RPD = 2.88 and 3.74; Different temperature (−14°C, −24°C, and −80°C): $R_{p}^{2}$ = 0.9151 and 0.9703, RPD = 2.28 and 3.8, respectively	Zhuang et al. (2023)
	Pork	480.8–1002.2 nm	carbonyl and sulfhydryl	PLSR	$R_{p}^{2}$ = 0.9275 and 0.9512, RMSEP = 0.0812 and 1.2979, respectively	Cheng et al. (2022)
	Beef	210–450 nm	Freshness	PLSR	Accuracy of 92.54% (calibration) and 86.96% (validation)	Liu et al. (2020)
	Beef	210–600 nm	Freshness	CA, EEM, and PARAFAC	Accuracy of 95.56% (calibration) and 93.33% (validation)	Liu et al. (2019)
	Beef	200–650 nm	Quality	PCA and PLSR	$R_{c}^{2}$ = 0.912, RMSEC = 0.488, $R_{p}^{2}$ = 0.903, RMSEP = 0.581	Liu et al. (2023)
	Beef	290, 322, 340 and 410 nm	Adulteration	PCA and PLSR	$R_{\mathrm{cv}}^{2}$ = 0.95, cross‐validated grouping success rate of 97%	Saleem et al. (2024)
	Frozen fish and fillet samples	250–800 nm	Freshness	PLSR	$R_{\mathrm{cv}}^{2}$ = 0.85 and 0.94, RMSECV = 0.1115 and 0.0734, respectively	ElMasry et al. (2016)
Terahertz spectroscopy	Pork	0.2–2.0 THz	Freshness	BP‐ANN and AdaBoost	*R*_*p*_ = 0.84, RMSEP = 0.0989	Qi et al. (2019)
	Beef	0–15 THz	MC and MC loss	PLSR	$R_{p}^{2}$ = 0.9646 and 0.9817, respectively	Ren et al. (2023)
	Fish	0.1–4.0 THz	Adulteration	SVM	Accuracy of 99.56% (prediction)	Hu et al. (2023)
	Fish, beef, chicken, and pork	0.6–1.4 THz	Classification of species	PCA‐SVM	Accuracy of 90%–96.67%	Yang et al. (2021)
	Pork sausage	0.1–3.5 THz	metal contaminations	PCA and DA	Accuracy of 98.3%–100%	Wang et al. (2019)
	Chicken	0.2–1.6 THz	Estriol and testosterone	Quantitative regression model	*R* ^2^ = 0.9744 and 0.9527, respectively	Zhao, Wang, et al. (2025)

Abbreviations: AdaBoost, adaptive boosting; BP‐ANN, back propagation‐artificial neural network; BPNN, back propagation neural network; CA, cluster analysis method; DA, discriminant analysis; EAA, essential amino acid; EEM, excitation–emission matrix; GA, genetic algorithm; LSTM, long short‐term memory; MC, moisture content; MSC, multiplicative scatter correction; MVN, multivariate normal sampling; PCA, principal component analysis; PLS‐DA, partial least‐squares discriminant analysis; PLSR, partial least‐squares regression; *R* ^2^, the coefficient of determination; $R_{c}^{2}$, the coefficient of determination for the calibration set; $R_{\mathrm{cv}}^{2}$, the coefficient of determination for the cross‐validation set; $R_{p}^{2}$, the coefficient of determination for the prediction set; RF, random forest; RF‐PLS, random frog‐partial least‐square; RMSE, root‐mean‐square error; RMSEC, root‐mean‐square error of calibration; RMSECV, root‐mean‐square error of cross‐validation; RMSEP, root‐mean‐square error of prediction; RPD, performance deviation ratio; SNV, standard normal variate transformation; SPA, successive projections algorithm; SVM, support vector machine; SVMR, support vector machine regression; UMP, uridine monophosphate; UVE, uninformative variable elimination; WHC, water‐holding capacity.

## 3 Imaging Techniques

### 3.1 Hyperspectral Imaging

**FIGURE 5:**
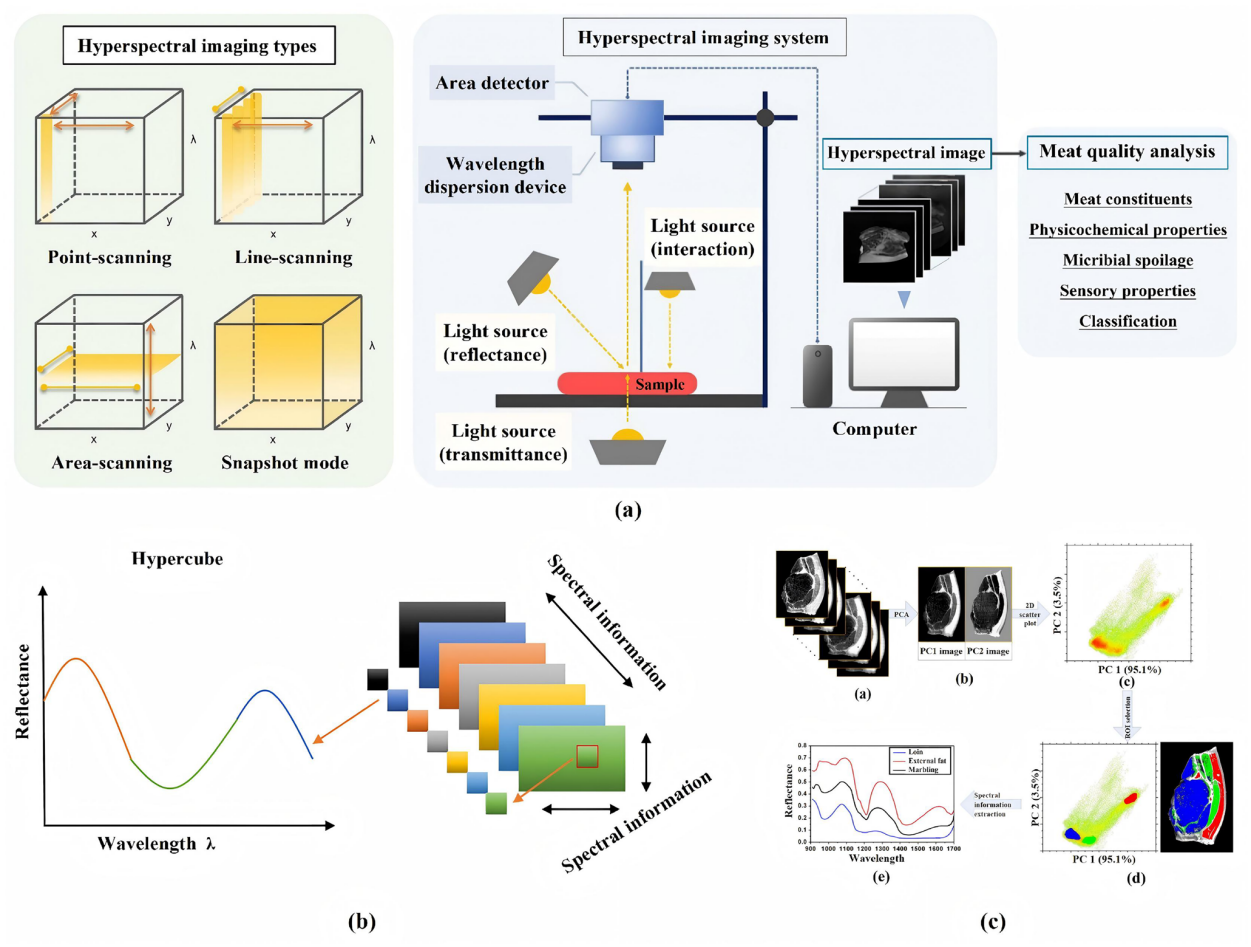
(a) System components and operating principles of an HSI system (Jo et al. 2024). (b) Graphical scheme of hypercube (Moharram and Sundaram 2023). (c) HSI combined with the PLS‐R method to obtain the distribution of drip loss in pork samples (Barbin et al. 2012).

HSI technology is an advanced optoelectronic detection fusion technique that can be designed to operate in various regions of the electromagnetic spectrum, including ultraviolet, visible, NIR, mid‐infrared, far‐infrared, and thermal infrared regions (Shi et al. 2021). This technology integrates spectral detection with digital computer vision, enabling the external characteristics of meat to be detected through image information and its internal quality characteristics to be assessed via spectral information. It is characterized by continuous multi‐band coverage, high spectral resolution, and the combination of spatial and spectral information in a single data cube (He and Sun 2015). The detection wavelength ranges from 400 to 1700 nm, covering both the visible and near‐infrared regions. Additionally, HSI can acquire spatial data on irregular samples, such as chemical composition, physical properties, quantity, and spatial location (Aheto, Huang, Tian, et al. 2019). The HSI system comprises a light source, an optical system, a sample fixation platform, and an interface computer (Ge et al. 2019), as illustrated in Figure 5a.

Hyperspectral images form a three‐dimensional (3D) data structure known as a “hypercube” (*x*, *y*, *λ*), where each pixel contains location‐specific spectral data that can be utilized to characterize the composition of a specific pixel (Khaled et al. 2021). Figure 5b presents the correlation between the spectral and spatial dimensions of a hyperspectral image. After processing the hyperspectral image data, samples can be quantitatively analyzed or labeled quantitatively based on spectral features, as shown in Figure 5c.

The HSI technique for detecting foodstuffs generally considers two wavelength ranges: 400–1000 and 900–1700 nm. Studies on meat and aquatic products predominantly focus on the 900–1700 nm range. Kamruzzaman et al. (2016) used VNIR‐HSI (400–1000 nm) and image processing techniques to detect ground beef adulterated with chicken at the pixel level. They found that the PLSR model was the most effective in discriminating adulteration. In practical applications, a comparison of the advantages and disadvantages of the 400–1000 and 900–1700 nm wavelength ranges for the detection of adulterated meat and meat products is possible. This can be done by testing samples with both wavelength ranges and comparing the results to determine the optimal range for best results (Sanchez et al. 2022).

HSI facilitates the visualization of various detection indicators (Cheng et al. 2023). However, hyperspectral images involve substantial data volumes and multiple dimensions, necessitating the use of robust and efficient algorithms for noise reduction, feature wavelength selection, and the establishment of a high‐precision discriminative model. This approach is critical for the utilization of hyperspectral analysis and constitutes the central focus of the present study (Kutsanedzie et al. 2019). Siripatrawan (2018) employed the HSI in conjunction with PLSR to evaluate the quality of vacuum‐packaged dry‐cured sausages. He employed the relationship between HSI hypercube data and the deterioration quality index (DQI) to plot the color distribution of the DQI and visually ascertain the relationship between different qualities and storage time. Bonah et al. (2020) performed rapid monitoring of foodborne pathogenic bacteria ( *Escherichia coli* O157 and *Staphylococcus aureus* ) contamination in fresh porcine longissimus muscle using visible near‐infrared (vis–NIR) HSI spectroscopy combined with the PLSR algorithm. This method offers the advantages of being nondestructive, simple, and fast, facilitating real‐time monitoring of food quality (Aheto et al. 2020).

HSI enables sensory attribute prediction, ingredient inspection, contaminant detection, foreign object recognition, and bacterial spoilage detection, which can improve consumer confidence in meat products (Khaled et al. 2021; Shi et al. 2023; Yang et al. 2023). Despite its advantages, HSI has certain limitations, including the generation of large three‐dimensional data volumes, redundancy in spectral band data, and high equipment costs. In the future, it will be crucial to explore low‐cost and high‐efficiency algorithms for effective dimensionality reduction. Additionally, there is a need for low‐cost, integrated inspection systems that combine sensors and computers to support quality inspection, process control, and pricing systems in food processing enterprises.

### 3.2 Multispectral Imaging

In recent years, MSI technology employing human‐machine interaction has witnessed substantial advancements across various agriculture domains, such as crop growth status monitoring (Prikaziuk et al. 2022), pest control (Chandel et al. 2021), and crop yield estimation (Vatter et al. 2022). MSI represents a simpleness of HSI by utilizing an optimized subset of wavelengths from HSI. As a result, MSI requires significantly less data for operation compared to HSI (Ma et al. 2016; Sendin et al. 2018). Unlike high‐resolution HSI systems, MSI systems select spectral channels that are sensitive to food‐related features. MSI acquires data in multiple discrete wavelength bands ranging from the ultraviolet to the NIR spectrum (200–2500 nm), thereby adding a new dimension of spectral information. The images obtained from MSI systems are referred to as 3D “hypercubes”, which can be described as each two‐dimensional (2D) pixel (*x* and *y*) associated with a one‐dimensional spectrum (*λ*) (Wang et al. 2021). The term “Hypercube” signifies a complex dataset, also referred to as a “data cube”, encompassing extensive information such as color, texture, and chemical composition (Ma et al. 2016). MSI is capable of capturing image data within a discontinuous spectral range, generating the characteristic wavelength of each pixel in the target object. This capability renders MSI highly suitable for applications in food quality analysis and visualization (Gomes et al. 2022).

**FIGURE 6:**
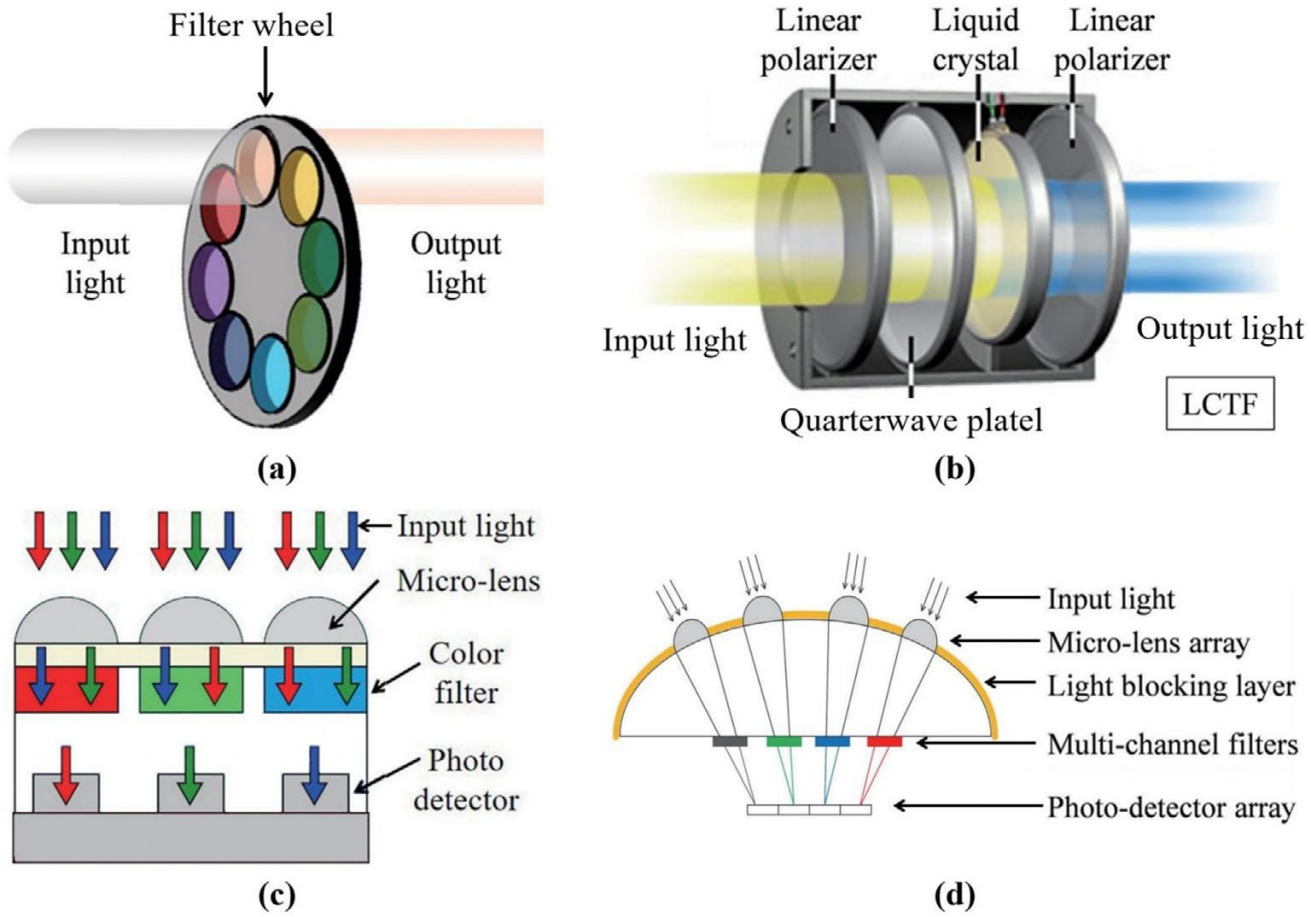
Multispectral imaging system based on a filter wheel (a) and an LCTF (b). Multispectral imaging based on integrated optical components: color filter array (c) or silicon nanowires (d). LCTF, liquid crystal tunable filter (Chen et al. 2021).

In conventional MSI systems, different wavelengths of light are typically separated via wavelength selection devices, such as a filter wheel or a liquid crystal tunable filter. Such systems are shown in Figure 6a,b and, respectively. However, this design is constrained by its inability to capture multiple spectral images simultaneously, thereby rendering the overall operation of the system time‐consuming and inefficient. To improve the practicality of the technology, researchers have been designing advanced compact MSI systems. As shown in Figure 6c,d and, these innovations incorporate color separation units (e.g., absorptive color filter array and silicon nanowires) with imaging sensors (Chen et al. 2021).

MSI has been acknowledged as a rapid, non‐invasive analytical technique with substantial potential for application in the quality and safety assessment of meat and meat products. Its application encompasses spoilage detection, adulteration identification, nutritional evaluation, microbiological analysis, as well as color and tenderness determination. In numerous instances, MSI is integrated with advanced data processing methods to enhance its efficacy. Omwange et al. (2021) employed MSI in conjunction with PLSR to assess the freshness of intact Japanese dace stored below 5°C. This method was demonstrated to be effective in nondestructively evaluating fish freshness by capturing images at varying excitation wavelengths. Ropodi et al. (2017) employed a combination of MSI with PLS‐DA and SVM to accurately detect adulterated ground horse meat in beef, achieving a classification accuracy of 95.31%. Tsakanikas et al. (2016) introduced a novel approach for visually assessing microbial contamination in meat samples using MSI. They developed a support vector regression (SVR) model to quantitatively estimate microbial counts during storage, achieving an accuracy range of 89.2%–80.8%.

Despite the success achieved with multispectral techniques in food detection, several challenges persist. The intrinsic spectral variability of diverse food products necessitates targeted optimization of detection methodologies. Furthermore, reducing the cost of multispectral techniques is critical to encourage broader implementation. In the future, the integration of multispectral techniques with other nondestructive testing methods may significantly enhance detection accuracy and comprehensiveness of detection (Khaled et al. 2021).

### 3.3 X‐Ray Imaging

X‐rays, also referred to as Roentgen rays, are a highly penetrating form of electromagnetic radiation with wavelengths ranging from 0.01 to 10 nm and photon energies ranging from 0.1 to 120 keV (Du et al. 2019; Huang and Liang 2024). Two types of X‐rays are distinguished: soft X‐rays with wavelengths ranging from 10 to 0.10 nm and photon energies of approximately 10 keV, and hard X‐rays with wavelengths between 0.10 and 0.01 nm and photon energies ranging from 10 to 120 keV (Takahara et al. 2020). Due to the high penetrative capacity of hard X‐rays, which can potentially contaminate foodstuffs, soft X‐rays are frequently employed for food monitoring.

**FIGURE 7:**
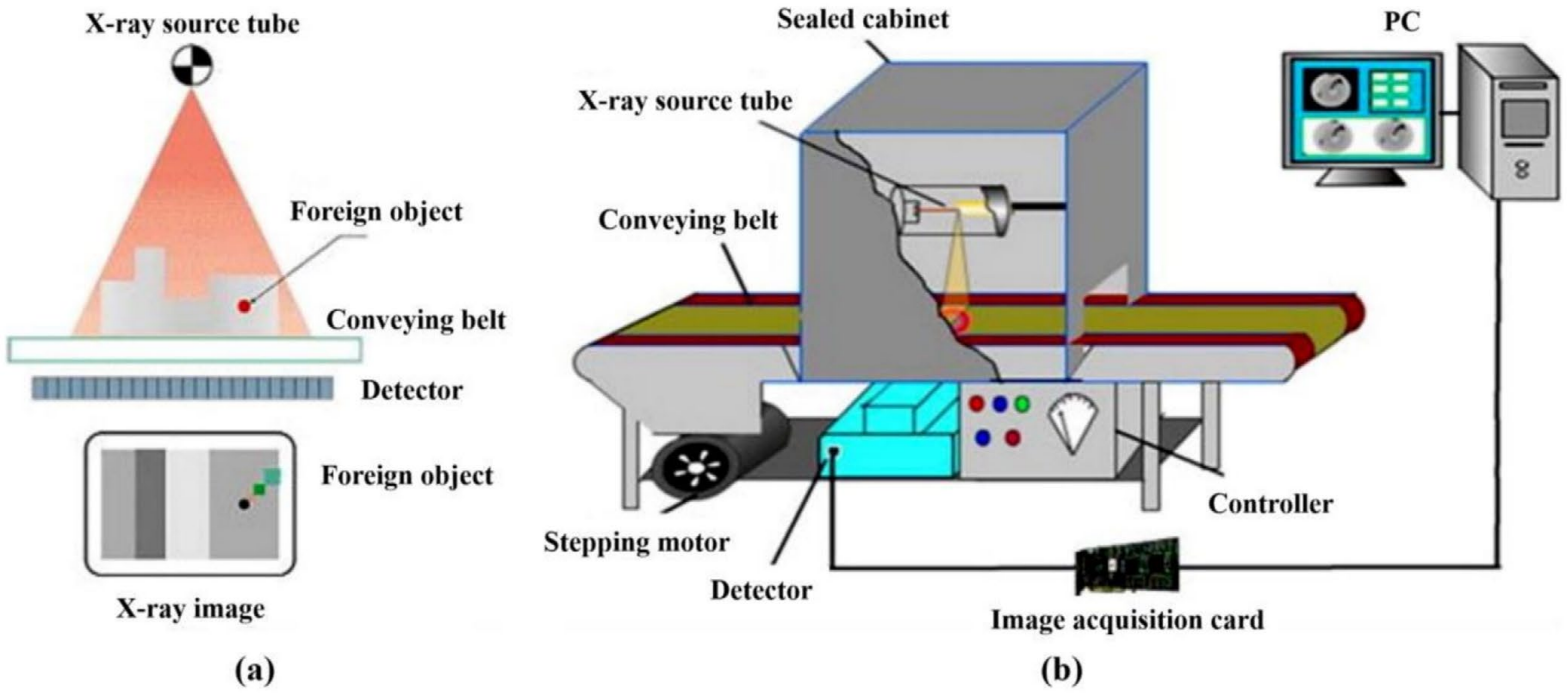
(a) Principles of soft X‐ray imaging. (b) System components and operating principles of the soft X‐ray inspection system (Chen et al. 2013).

The principle of soft XRI detection is shown in Figure 7a. When X‐rays penetrate a meat product, differences in internal density and thickness cause varying degrees of X‐ray absorption. As a result, the intensity of X‐rays in the penetrated portion changes, providing information about the interior of the object. As X‐rays are emitted from the product, they are detected by the sensor, which transforms the energy signal into a visual representation of the product's interior. Foreign materials appear as darker shades of gray, facilitating the identification of the foreign contaminant, as shown in Figure 7b. The soft X‐ray inspection system is composed of an X‐ray generator, a line scan sensor, a conveyor belt, a stepper motor, an image acquisition card, and a computer (Chen et al. 2013).

XRI, particularly X‐ray computed tomography (CT), is a non‐invasive technique that allows for the visualization of the interior composition of objects. Its applications include the monitoring of the internal composition of meat, such as the percentage of lean meat (Börgeson et al. 2024) and the content of intramuscular fat (Anderson et al. 2018). Gilles (2015) used CT technology to assess the composition and quality of pig carcasses. This technology facilitated the determination of the proportions of the three primary tissues (fat, lean, and bone) and the subsequent grading of the carcasses based on the thickness of the fat and lean layers. Gardner et al. (2018) performed DEXA (dual‐energy x‐ray absorptiometer) scans on 607 lamb carcasses obtained from seven abattoirs and subsequently utilized CT techniques to ascertain the proportions of fat, lean, and bone. Validation tests across datasets showed the remarkable precision of the predictions. López‐Campos et al. (2018) employed dual‐energy X‐ray absorptiometry (DXA) in conjunction with PLSR to predict the total amount of lean, fat, and bone in beef carcass halves and major cuts. The model demonstrated high accuracy in predicting tissue weights throughout the carcass halves. The *R* ^2^ of lean, fat, and bone were 0.991, 0.985, and 0.941, respectively. These studies demonstrate the potential of XRI for the visualization and monitoring of internal meat composition.

A comparison of XRI and MRI is frequently made due to their similarity in application areas. In comparison to MRI, soft XRI is comparatively inexpensive to utilize and has high usability and few material limitations. Based on sample density, XRI has advantages in detecting internal tissues and external contamination in meat, but struggles to detect a wide range of foreign matter. Generally speaking, XRI technology has difficulty recognizing foreign objects with a density similar to that of water. Such objects include, but are not limited to, hair, paper, and plastics. Moreover, there are still limitations in practical applications (Sanchez et al. 2020).

### 3.4 Thermal Imaging

**FIGURE 8:**
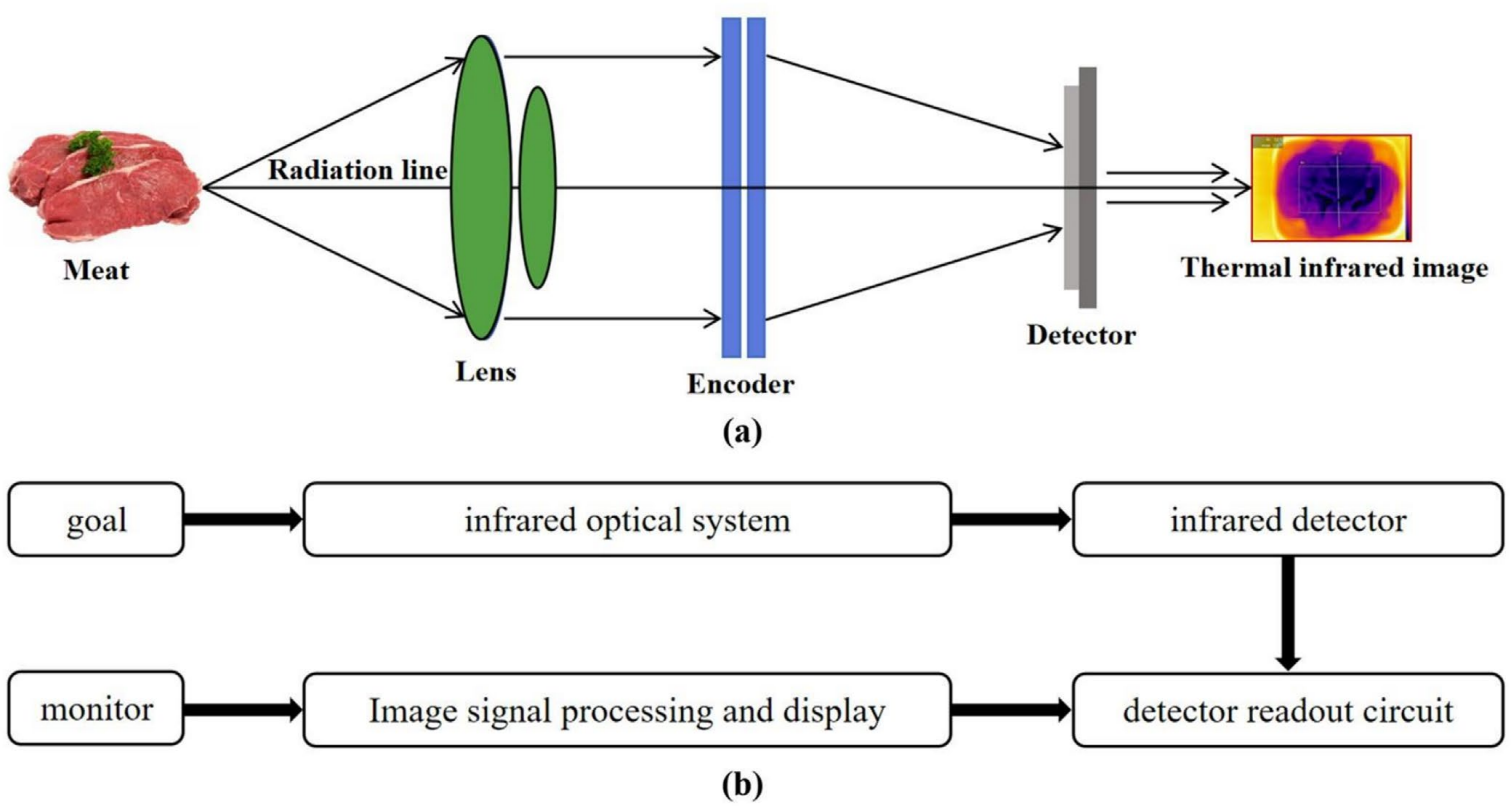
(a) Experimental setup of TI for meat inspection. (b) The working process of TI for meat inspection.

TI technology was developed on the basis that all objects emit infrared light above absolute zero (−273°C) (Zhang et al. 2022). TI systems typically include a camera, an optical system, a detector array, and a signal and image processing system, as shown in Figure 8a. The TI cameras convert the infrared energy emitted by the target into an electrical signal through an infrared detector and display it as a monochrome or color thermal image, as shown in Figure 8b. Given that the intensity of infrared energy emitted by an object is directly proportional to the fourth power of its surface temperature, the intensity of this energy will increase as the temperature rises. The conversion of infrared energy intensity to image color facilitates the extraction of the spatial temperature distribution map of the object scene, thereby enabling the determination of the measurement target's nature (Yang et al. 2019).

Unlike RGB technology, TI works independently of external light sources, detecting only the infrared radiation emitted by an object. This capability enables more expeditious and effective detection of thermal changes on the surface of the target object. Furthermore, TI systems possess the capability to detect radiation from short‐wave to long‐wave infrared. The varying wavelengths of infrared radiation exhibit different sensitivities within distinct temperature range intervals (Riaz et al. 2023). This attribute has led to TI systems becoming the preferred detection method for most food processing facilities.

During food processing, practices like high‐temperature sterilization and low‐temperature storage are key to ensuring product quality and flavor. In the food industry, thermocouples (Kor and Icier 2016), thermometers (Elshahat et al. 2019), and thermistors (Matvienko et al. 2020) are frequently employed for temperature monitoring. However, these instruments occasionally necessitate intrusive measurements, which have the potential to induce complications such as cross‐contamination. Conversely, TI can rapidly assess food indicators without the necessity of direct contact. Zheng et al. (2021) employed thermal imaging and CNN to classify and quantify minced lamb adulterated with pork. The results demonstrated that the accuracy of the optimal characterization model was 99.99%, and thermal imaging was found to be more cost‐effective and accessible than other detection methods, such as HSI and high‐performance liquid chromatography (HPLC). Consequently, this method is regarded as both economical and convenient in the domain of food detection. Wang, Zhu, et al. (2023) employed thermal imaging and CCD imaging in a collaborative approach to detect lamb adulteration, subsequently developing a classification and prediction model through the integration of these two images. The findings indicated that the most accurate classification model exhibited a precision rate of 99.30%. Consequently, the synergistic integration of thermal imaging and CCD imaging has emerged as a novel and promising tool for the detection of adulteration in mutton and other food products.

The presence of microorganisms and foreign objects in meat constitutes a significant food safety concern. TI technology differentiates foreign objects from food products by capturing thermal images of foods and foreign objects with high contrast. Lipińska et al. (2022) employed a TI camera with an uncooled microbolometer heat detector to detect *Bacillus subtilis* ATCC6633 bacteria in meat, and it was found that the TI measurements used were capable of differentiating between contaminated samples (minimum 10^6^CFU/g) and sterile samples. This finding indicates that the TI technique can detect microorganisms in meat.

TI technology holds great potential for meat quality and safety detection, enabling the determination of meat freshness and spoilage risk through the analysis of temperature changes. However, practical applications of this technology are encumbered by significant challenges. The technology can be categorized into two distinct methods: passive and active. Passive technology relies on the detection of thermal radiation emitted by the object itself, whereas active technology necessitates the application of an external thermal excitation. Integrated systems for active thermal imaging measurements incorporate heating or cooling devices to create thermal differences, thereby enhancing image contrast and improving detection accuracy. However, given the high sensitivity of most foods to changes in ambient temperature, the heating/cooling process may have a deleterious effect on the food. Consequently, the temperature tolerance characteristics of meat must be given due consideration (Gowen et al. 2010). Furthermore, the equipment necessary for the operation of thermal imaging systems is costly, and environmental thermal interference poses a significant obstacle to the development of industrial‐grade thermal imaging sensors. Table 2 lists some typical examples of the use of imaging technologies for meat quality and safety testing in recent years.

**TABLE 2:** Summaries of different imaging techniques applied for meat quality and safety detection.

Techniques	Samples	Testing parameters	Measured attributes	Chemometric procedures	Performance	References
Hyperspectral imaging	Pork	400–1600 nm	TBC and VBN	PLSR	$R_{p}^{2}$ = 0.7583 and 0.8441, respectively	Choi et al. (2024)
	Pork	900–1700 nm	Fat	MLR	$R_{c}$ = 0.96, $R_{\mathrm{cv}}$ = 0.96, $R_{\mathrm{fv}}$ = 0.96	Huang et al. (2017)
	Pork	400–1000 nm	Intramuscular fat	SVM, SG, SNV, MSC and PLSR	$R_{p}^{2}$ = 0.9635, RMSEP = 0.885	Ma et al. (2018)
	Pork	400–800 nm	pH	SVR	Training set: accuracy of 88.02%, $R^{2}$ = 0.9343; Test set: accuracy of 87.50%, $R^{2}$ = 0.9318	Yao et al. (2019)
					$R_{p}^{2}$	
	Beef	400–1600 nm	TBC and VBN	PLSR	$R_{p}^{2}$ = 0.74 and 0.81, RMSE = 0.63 and 0.93, respectively	Ismail et al. (2025)
	Beef	496–1000 nm	Adulteration	LS‐SVM	$R_{c}^{2}$ = 0.97, RMSEC = 0.0474, $R_{p}^{2}$ = 0.95, RMSEP = 0.0567	Zhao et al. (2019)
	Beef	400–1000 nm	TVC	PLSR RF, CA and LS‐SVM	Accuracy of 97.14%	Yang et al. (2017)
	Lamb	400–1000 nm	Adulteration	SPA and SG	$R_{p}^{2}$ = 0.995, RMSEP = 0.0251	Zheng et al. (2019)
	Chicken	400.68–1001.61 nm	Soybean protein	VGG16‐SVM	Accuracy of 99.1% (training set) and 98.1% (test set)	Sun et al. (2024)
	Chicken	380–1020 nm	WB severity	PLSR, RC and LDA	$R_{p}$ = 0.915, RMSEP = 2.26, Accuracy of 97.22% (calibration) and 91.67% (prediction)	Liu et al. (2025)
	Fish	950–1650 nm	Classification	PLS‐DA	$R_{c}^{2}$ = 0.9547	Park et al. (2024)
	Fish	400–1000 nm	Moisture	PLSR	$R_{c}^{2}$ = 0.9416, RMSEC = 0.0455, $R_{\mathrm{cv}}^{2}$ = 0.9278, RMSECV = 0.0512, $R_{p}^{2}$ = 0.9117, RMSEP = 0.0563	Qu et al. (2017)
Multispectral imaging	Pork	405–970 nm	Bone fragments	SPA, LDA and PCA–LDA	Accuracy of 100% (test set)	Wang, Zhang, et al. (2021)
	Beef and horsemeat	405–970 nm	Adulteration	PLS‐DA, RF, and SVM	Accuracy of 95.31%	Ropodi et al. (2017)
	Beef	500–800 nm	Classification	LDA	Accuracy of above 90%	Li, Li, et al. (2021)
	Chicken	405–970 nm	TVC and Pseudomonas spp	PLSR	RMSE = 1.359 and 1.574	Spyrelli et al. (2021)
	Chicken	350–950 nm	TVC and LAB	PLSR	Cooked turkey breast: $R_{c}^{2}$ = 0.92 and 0.89, $R_{\mathrm{cv}}^{2}$ = 0.84 and 0.78, $R_{p}^{2}$ = 0.93 and 0.9; Smoked turkey breast: $R_{c}^{2}$ = 0.79 and 0.89, $R_{\mathrm{cv}}^{2}$ = 0.82 and 0.94, $R_{p}^{2}$ = 0.82 and 0.98, respectively	Govari et al. (2025)
	Fish	220–600 nm	Freshness	PLSR	$R_{p}^{2}$ = 0.94, RMSE = 0.0242	Omwange et al. (2021)
X‐ray imaging	Pork	140 kV and 145 mA	LMP	PLSA and irPLSR	$R_{c}$ = 0.99 and 0.98, $R_{p}$ = 0.96 and 0.96, RMSEC = 0.63 and 0.72, RMSEP = 0.96 and 0.86, respectively	Mishra and Font‐i‐Furnols (2024)
	Beef	100 kV and 180 mA	Lean %, fat % and bone %	PLSR	RMSE = 0.0231, 0.0251 and 0.0112, respectively	Calnan et al. (2021)
	Beef	120 kV	Fat	ANOVA	*R* = 0.9	Holló et al. (2018)
	Beef	100 kV and 0.188 mA	Water and lipid	Simple and multiple linear regressions	*R* ^2^ = 0.97, RMSE = 0.008, rCV = 0.0072	Xavier et al. (2023)
	Lamb	110 kV and 150 mA	Fat %	PLSR	$R_{p}^{2}$ = 0.91, RMSEP = 0.0132	Connaughton et al. (2020)
Thermal imaging	Pork and mutton	7.5–14 μm	Adulteration	CNN	Accuracy of 99.97% (validation) and 99.99% (test), $R_{c}^{2}$ = 0.9933, RMSEC = 0.0251, $R_{p}^{2}$ = 0.9933, RMSEC = 0.0252	Zheng et al. (2021)
	Lamb	7.5–14 μm	Adulteration	Deep residual network	Accuracy of 99.3%, Pork proportions: $R_{p}^{2}$ = 0.9717, RMSEP = 0.0238, RPD = 7.8696; Duck proportions: $R_{p}^{2}$ = 0.9616, RMSEP = 0.0277, RPD = 5.1015	Wang, Zhu, et al. (2023)
	Chicken	8–14 μm	Pathological phenomena	YOLOv8	Accuracy of 98.8%	Elmessery et al. (2023)

Abbreviations: 1D‐CNN, one‐dimensional convolutional neural network; ANN, artificial neural network; ANOVA, analysis of variance; CA, correlation analysis; CNN, convolution neural networks; FM, foreign material; irPLSR, iterative reweighted PLSR; LAB, lactic acid bacteria; LDA, linear discriminant analysis; LMP, lean meat percentage; LS‐SVM, least‐squares support vector machine; MLR, multiple linear regression; MSC, multiplicative scatter correction; PCA, principal component analysis; PLS‐DA, partial least‐squares discriminant analysis; PLSR, partial‐least‐squares regression; $R^{2}$, the coefficient of determination; $R_{c}^{2}$, the coefficient of determination for the calibration set; $R_{\mathrm{cv}}^{2}$, the coefficient of determination for the cross‐validation set; $R_{p}^{2}$, the coefficient of determination for the prediction set; RC, regression coefficient; rCV, the residual coefficient of variation; RF, random forest; RMSE, root‐mean‐square error; RMSEC, root‐mean‐square error of calibration; RMSECV, root‐mean‐square error of cross‐validation; RMSEP, root‐mean‐square error of prediction; RPD, performance deviation ratio; SG, Savitzky–Golay; SNV, smoothing, standard normal variate; SPA, successive projections algorithm; SVM, support vector machine; SVR, support vector regression; TAB, total aerobic bacteria; TBC, total bacterial count; TVC, total viable counts; VBN, volatile basic nitrogen; WB, wooden breast.

## 4 Data Analysis Methods Combining Deep Learning

The advent of artificial intelligence (AI) signifies a transformative paradigm shift, heralding an era defined by data‐driven decision‐making and innovation (Zareef, Arslan, Hassan, Ahmad, et al. 2021). The diverse technological approaches and interdisciplinary integration capabilities of these systems have been shown to enhance the accuracy, speed, and nondestructive nature of evaluations. The primary function of these AI tools is to facilitate multidimensional information fusion assessments of meat quality and safety through data collection, processing, and modeling (Zhang et al. 2025).

The utilization of artificial intelligence‐driven machine learning (ML) and DL methodologies has been demonstrated to markedly enhance the efficiency of processing high‐dimensional data. In the domain of meat quality and safety inspection, multidimensional data fusion methodologies based on ML techniques emerge as a pivotal solution for enhancing the processing efficiency of complex datasets and refining the universality of detection models. This approach uses multiple data patterns to analyze the chemical, physical and biological properties of meat products in a comprehensive way. This boosts the efficiency and accuracy of tasks such as quality inspection, component analysis and monitoring spoilage and contamination (Liu, Liu, et al. 2024). Examples of common ML algorithms include SVM, RF, PCA, LDA, and ANN. Each algorithm is suited to specific scenarios: SVM excels at handling small samples and high‐dimensional data (Xu et al. 2010); RF is ideal for analyzing multimodal data (Geng et al. 2022); and PCA and LDA are well‐suited to processing high‐dimensional data (Sajjad et al. 2025). Therefore, selecting the appropriate algorithm is essential for meeting specific detection requirements. ML models are frequently combined with spectral and imaging technologies for the application of rapid, nondestructive meat quality and safety analysis. Specific case studies are presented in Tables 1 and 2.

DL, a subset of ML, refers to a neural network with multiple layers that autonomously extracts complex features from images (Wang, Yang, et al. 2024). It is currently employed in a wide range of applications, including freshness detection (Huang et al. 2025; Kozan and Akyürek 2024), ingredient analysis (Shen et al. 2024; Zhou et al. 2020), and foreign object recognition (Campos et al. 2023; Pan et al. 2023). DL can effectively leverage both spatial and spectral information. Several recent studies have successfully integrated DL with spectroscopic techniques (Zhao, Adade, et al. 2025) or imaging techniques (Khan et al. 2025) for meat quality and safety testing. These studies demonstrate that such integration significantly enhances the accuracy and efficiency of assessment, thereby providing more reliable information to consumers and producers (Menezes et al. 2025). Conventional chemometrics and ML approaches rely on handcrafted models based on domain knowledge and assumptions and may thus be constrained or prone to errors (Huang et al. 2025). Nevertheless, DL exhibits superior capacities in processing large‐scale, high‐dimensional data, such as hyperspectral images, showcasing advanced data processing and analytical abilities (Soni et al. 2021).

By combining HSI with advanced DL techniques, such as convolutional neural network (CNN) and generative adversarial network (GAN), deep features can be effectively extracted from high‐dimensional HSI data, thereby improving the accuracy of meat quality and safety classification and detection tasks (Bera et al. 2022; Campos et al. 2023; Khan et al. 2025). Campos et al. (2023) proposed a novel semi‐supervised hyperspectral DL model based on GAN, utilizing a modified one‐dimensional U‐Net as a discriminator to detect foreign objects on raw chicken breast slices, achieving an accuracy of 100%. Syed et al. (2024) employed a combination of HSI and DL techniques to detect parasitic infections in whitefish, achieving a final detection rate of 73%, which significantly surpasses the 50% achieved by conventional manual examination. Al‐Sarayreh et al. (2020) introduced a novel deep 3D convolutional neural network (3D‐CNN) model for classifying meat in HSI images, achieving overall accuracies of 96.9% for NIR and 97.1% for visible snapshot HSIs, representing a substantial improvement in accuracy. Furthermore, 3D‐CNN‐enabled HSI has been utilized to assess lipid oxidation in pork, enabling nondestructive monitoring of oxidative damage in pork (Cheng et al. 2023). Concurrent studies have also integrated DL with NIRS (Albano‐Gaglio et al. 2025), RS (Li, Sheng, et al. 2024), FS (Fan et al. 2024), and other methods for detecting meat quality and safety.

**FIGURE 9:**
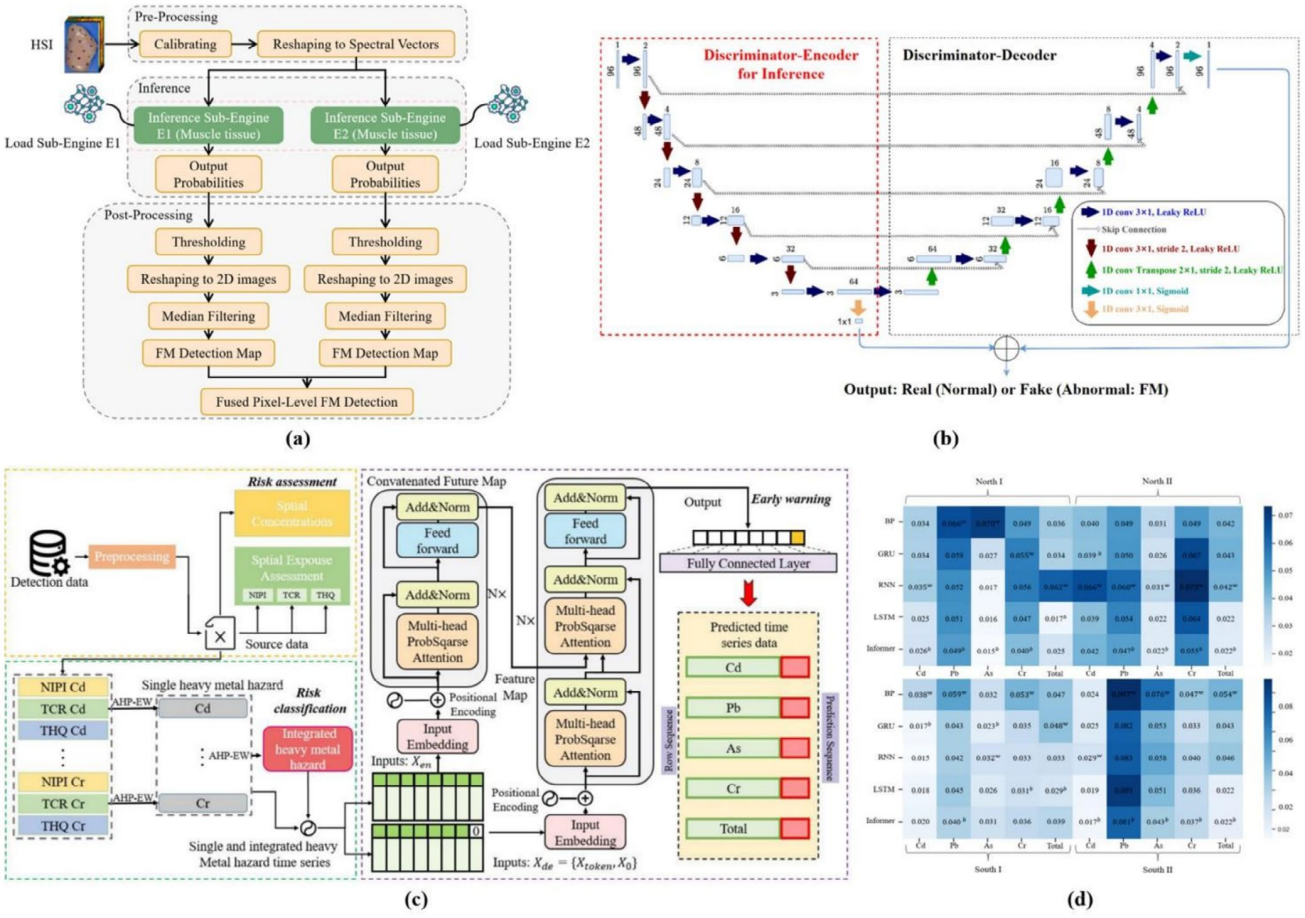
(a) Inference pipeline for FM detection based on HIS and DL. (b) Two pre‐trained GAN discriminators in an inference model (Khan et al. 2025). (c) A general framework for heavy metal detection in meat based on a transformer model. (d) The root‐mean‐square error (RMSE) of different models in a heavy metal dataset. ^b^Best results in the dataset; ^w^Worst results in the dataset (Wang, Gao, et al. 2023).

DL has significantly reshaped the field of meat quality and safety assessment, yet it still faces numerous challenges. As this technology continues to evolve, researchers have actively explored solutions to address the existing limitations. A key challenge in the current domain is the lack of large, standardized datasets, which directly affects the performance of DL models. Naturizal et al. (2024) developed a CNN‐based chicken freshness detection system that categorizes chicken meat into fresh, less fresh, and rotten, and the system showed 90% accuracy in testing. However, the reliance on pre‐trained model weights leads to increased memory requirements and computational demands. To enhance efficiency, DL models should be integrated into an embedded hardware system (Rokh et al. 2023). Studies have demonstrated that model compression effectively reduces the size and computational complexity of DL models. Additionally, field‐programmable gate arrays (FPGAs) facilitate the development of customized hardware configurations, thereby accelerating the inference process (Khan et al. 2025). As shown in Figure 9a, Khan et al. (2025) enhanced the efficiency of DL inference for foreign object detection by leveraging hardware acceleration techniques. The inference model comprises two pre‐trained GAN discriminators, as shown in Figure 9b. The findings indicate that this technique reduces inference time by a factor of five and decreases model size by 50% compared to the conventional method, while maintaining high detection accuracy.

DL is increasingly recognized as a valuable and indispensable tool in meat quality and safety detection, as shown in Table 3. However, due to the inherent intricacy of DL models, they are frequently viewed as black boxes, underscoring the critical need for interpretability. Various interpretable methods, both local and global, are utilized in food authenticity assessment, often incorporating visualizations. Despite these advancements, model accuracy remains paramount, and research into interpretability is still in its nascent stages. Future research should prioritize the design of self‐interpreting models, the achievement of real‐time interpretability, the establishment of unified evaluation metrics, the integration of interpretability into evaluation frameworks, and the utilization of advanced generative pre‐training models.

**TABLE 3:** Summaries of different DL techniques applied for meat quality and safety detection.

Category	Measured attribute	Model	Performance	References
Beef, lamb, pork, and other red meats	Classification	3D‐CNN	Accuracy of 96.9%	Al‐Sarayreh et al. (2020)
Pork	Microbial	CNN and DCRNet	Accuracy of 81.2%	Zheng et al. (2024)
Pork	gel strength and whiteness	CNN‐LSTM	$R_{p}^{2}$ = 0.9515 and 0.9383, respectively	Li, Sheng, et al. (2024)
Pork	Classification	GAN	Accuracy of 89%	Pan et al. (2023)
Beef	Marbling	Unet++, Deeplabv3+ and SegFormer	Accuracy of 90.85%	Cai et al. (2024)
Beef	Adulteration	Swin‐T	Accuracy of 97.5%	Gao et al. (2024)
Chicken	Monstrosity	GAN	Accuracy of 100%	Campos et al. (2023)
Chicken	gel strength	CNN and LSTM	$R_{p}^{2}$ = 0.9582 and 0.9882, respectively	Nunekpeku et al. (2025)
Fish	Drone	DNN	Accuracy of 73%	Syed et al. (2024)
Fish	Classification	FishNET‐S and FishNET‐T	Accuracy of 84.1% and 68.3%, respectively	Jayasundara et al. (2023)
Fish	Classification	CNN	Accuracy of 95%	Tupan et al. (2025)

Abbreviations: CNN, convolutional neural network; DCRNet, Duplex Contextual Relation Network; DNN, deep neural network; GAN, generative adversarial network; LSTM, long short‐term memory; Swin‐T, Swin Transformer.

Among various AI techniques, large‐scale language models (LLMs), including the generative pre‐trained transformer (GPT) family, have garnered substantial attention due to their profound influence on data analysis and interpretation. LLMs leverage deep neural networks with multiple attention mechanisms, showcasing superior model complexity, larger data volumes, and enhanced computational capabilities compared to alternative models (Ma et al. 2024). In the context of meat quality and safety testing, LLMs are capable of identifying meat components (Zheng et al. 2024) and harmful substances (Wang, Gu, et al. 2024) from meat images or descriptions, as well as estimating nutritional content. Their applications extend across domains such as nutritional analysis, meat safety, supply chain management, and consumer behavior analysis (Ma et al. 2024). Wang, Gao, et al. (2023) constructed a multi‐step food contaminant risk prediction model based on the transformer framework, as depicted in Figure 9c. The prediction results of the model, presented in Figure 9d, demonstrate that the transformer model surpasses other models in detecting heavy metals in meat products across 14 datasets. Looking ahead, this technology could be integrated with augmented reality (AR) to develop a predictive model for meat quality and safety, aiding consumers in assessing rawness, freshness, and analyzing visual characteristics such as color and texture. The model would predict sensory attributes like tenderness and juiciness, ultimately recommending appropriate meat products for specific consumer groups.

In the future, research into AI for assessing the safety and quality of meat will continue to focus on developing more advanced machine and DL algorithms. Integrating novel sensors and MSI technologies will enable more precise, real‐time, nondestructive detection. Simultaneously, enhancing the interpretability and robustness of AI models, and addressing data privacy and standardization challenges, will be crucial for the widespread adoption of AI in this field.

## 5 Challenges and Trends

Extensive research has shown that emerging techniques achieve precision rates exceeding 80%. However, the transition of these techniques from laboratory settings to industrial applications and ultimately to general consumer use continues to face numerous challenges. In spectroscopic techniques, parameters such as computational speed, scanning time, and sensor‐object distance play a pivotal role in the development of a real‐time automated processing system. Concurrently, addressing challenges such as suboptimal lighting conditions, noise effects, high hardware costs, and extensive data processing demands is critical. For instance, the accuracy of optical methods is substantially influenced by illumination conditions, and interference from existing light sources can undermine the precision and reliability of the optical system. A major barrier is the high cost of the technology, which impedes its widespread adoption. Furthermore, the extensive amount of data generated by imaging technologies often exhibits weak correlations with meat characterization, complicating effective processing. Additionally, current image processing algorithms fall short of meeting the stringent requirements for efficiency and accuracy in modern food manufacturing. The processing of large‐scale data streams presents a significant challenge to these algorithms, whose limited integration with specialized hardware often leads to extended testing times.

The performance of DL models is heavily contingent upon the quality and quantity of the training data. However, the paucity of large‐scale, standardized datasets in the domain of meat quality and safety detection restricts model generalizability (Arora and Mangipudi 2021). Moreover, deploying DL models on embedded hardware systems is essential for enabling real‐time assessment of meat quality and safety. Nonetheless, immediate attention is required to address challenges related to model compression and hardware acceleration (Khan et al. 2025). Meat quality and safety are influenced by a wide array of complex factors. The application of emerging spectroscopic, imaging, and DL technologies is predominantly restricted to specific domains, thereby limiting the capacity to simultaneously acquire diverse types of information for a comprehensive evaluation of meat quality and safety. The advantages, disadvantages, and application scenarios of emerging spectroscopy and imaging techniques in meat quality and safety detection are shown in Table 4. Research has demonstrated that the development of simple yet effective data fusion algorithms and integrated systems can leverage the synergistic effects of these analytical techniques, surpassing the performance of standalone methods. This enables the integration and fusion of data across multiple attributes, thus enhancing the accuracy of predictions regarding meat quality and safety (Jiang et al. 2021).

**TABLE 4:** Comparison of emerging spectroscopy and imaging techniques.

Techniques	Advantages	Disadvantages	Application scenarios
Near‐infrared spectroscopy	No complex pretreatment required, simultaneous analysis of multiple components, supports online real‐time monitoring, and strong penetration capability	Limited discrimination capability, complex model calibration, reliance on large sample volumes, susceptibility to interference from sample surface conditions, and difficulty in detecting heterogeneous samples	Quantitative analysis of nutritional components, monitoring of freshness and spoilage status, and identification and quantification of adulteration
Raman spectroscopy	High sensitivity and specificity, suitable for high‐moisture meat analysis, enables real‐time detection, and can be combined with a microscope for micrometer‐level micro‐area analysis	Prone to interference from the natural fluorescence of biomolecules, weak signal intensity, high cost of high‐sensitivity equipment, and stringent requirements for sample homogeneity	Quality indicator prediction, detection of chemical contaminants such as antibiotics, meat species identification and adulteration detection, and early warning of microbial spoilage
Fluorescence spectroscopy	Highly sensitive, rapid signal acquisition, real‐time monitoring capability, suitable for detecting microorganisms, and their metabolic products	Strong interference from natural fluorescent backgrounds, significant challenges in analyzing complex samples, high susceptibility to environmental factors, and difficulty distinguishing substances sharing similar fluorescent properties	Nutritional component assessment, rapid screening for biological contaminants, ATP detection, and detection of adulterants with fluorescent properties
Terahertz spectroscopy	Highly sensitive to moisture, provides information on the macroscopic and microscopic structure of meat, and exhibits characteristic absorption fingerprints	Limited penetration depth, high equipment costs, complex algorithm processing required, and stringent environmental requirements	Moisture‐related testing, texture and other characteristic analysis, meat microbiological, and foreign object detection
Hyperspectral imaging	High spectral resolution, rich information content, and integration of spatial and spectral data	Complex data analysis, high equipment costs, slow data collection speed, and challenges in real‐time applications	Quality assessment, adulteration detection, and biomedical imaging
Multispectral imaging	Moderate data volume, relatively simple equipment with low cost, excellent real‐time performance, and strong targeting capabilities	Low spectral resolution, limited information content, sensitive to matrix variations, and unable to analyze fine substances	Industrial online detection and rapid screening for specific substances
X‐ray imaging	High penetration, high resolution, and mature technology	Risks of ionizing radiation, low contrast for organic substances, inability to provide chemical information, and high equipment costs and large size	Detection of foreign objects within meat products and safety inspections
Thermal imaging	Displays temperature information, operates 24 h a day, and provides rapid detection and monitoring	Can only detect surface temperature, cannot obtain internal chemical information, susceptible to external environmental interference, and insensitive to color and texture	Monitoring of meat cold chain integrity, detection of abnormal hot spots, early spoilage screening, and production line temperature monitoring

Abbreviation: ATP, adenosine triphosphate.

Advancements in optical imaging, the Internet of Things (IoT), computer technology, and large‐scale language modeling hold considerable potential for reducing the cost of inspection equipment while improving the efficiency of data processing. Online inspection systems for meat, especially in production line settings, represent an inevitable trend in the industry. Emerging hardware technologies demonstrate considerable application potential in the domain of meat quality and safety inspection. Portable and handheld optical devices, particularly miniature spectrometers and handheld imaging systems, facilitate swift on‐site inspections and provide significant value at all stages of meat production and distribution (Zhai et al. 2021).

Miniature spectrometers are defined as compact spectroscopic analysis devices that integrate optical, mechanical, and electronic technologies. The primary advantages of these systems are portability, low cost, and rapid analytical capabilities (Yao et al. 2019). These instruments generally utilize NIRS technology for the nondestructive analysis of meat products. Portable NIRS spectrometers have been extensively adopted for on‐site quality control in the meat industry (Kademi et al. 2018). Portable imaging systems, particularly HSI systems, have the capacity to concurrently capture both spectral and spatial information of meat products, thereby providing more comprehensive data for the purpose of meat inspection. These techniques have been extensively employed in the detection of meat adulteration and the authentication of meat products (Yu et al. 2025). In addition to the development of hyperspectral imaging, other imaging technologies are also undergoing advancement. For instance, portable micro‐plasma emission spectrometers (μPD‐OES) combined with ML have been deployed for on‐site assessment of food freshness and adulteration detection (Asharindavida et al. 2023). However, current progress is hindered by several factors, including camera quality, high costs, and stringent operational requirements.

Furthermore, the rapid proliferation of IoT devices has led to a significant increase in the volume of data that requires real‐time processing (Qian 2025). Traditional cloud computing models are confronted with considerable challenges, including high latency, bandwidth limitations, and suboptimal scalability, when attempting to address these demands (Verma et al. 2023). Edge computing, as an emerging computing paradigm, has been demonstrated to significantly reduce data transmission latency and enhance processing efficiency by bringing computation and data storage closer to the source of data generation (Zhang and Fan 2024). This is of particular importance in applications requiring real‐time feedback, such as the monitoring of meat freshness, the instant detection of harmful substances, and quality control during meat processing (Zhang, Yang, et al. 2024; Zhang, Luan, et al. 2024). However, edge devices typically possess limited computational resources, and the efficient execution of complex AI models within these constraints remains an active research area (Aishah 2024; Damsgaard et al. 2024). Future research directions may include the development of more compact and robust hardware devices (Beitollahi et al. 2024), as well as the optimization of data processing algorithms to adapt to the resource limitations of edge environments (Aishah 2024).

Despite the potential of real‐time monitoring technologies for meat quality and safety, the necessity for labor‐intensive calibration procedures before use may impede practical implementation. The design of high‐performance, low‐cost online equipment for assessing meat quality and safety represents a critical direction for future technological advancement. Future research should emphasize the integration of multiple information sources and detection technologies to establish efficient real‐time meat detection systems.

Advancements in technologies such as spectroscopy, imaging, and artificial intelligence, in conjunction with the implementation of chemometrics methods, are effectively integrating academic research with industrial applications. This integration is propelling the development of meat safety and quality monitoring technologies. These technologies enhance the speed and precision of detection, thereby reducing costs and enabling real‐time monitoring. Consequently, consumers benefit from safer and higher‐quality meat products. The integration of smart sensing, wireless communications, and the Internet of Things (IoT) has the potential to expand the range of applications for food safety monitoring. Future research should concentrate on three things: DL, enhanced spectroscopy techniques, and the integration of multimodal technology with artificial intelligence solutions. Although the review focuses on meat quality and safety, the principles discussed herein can be broadly applied to other areas of food analysis.

## 6 Conclusions

This paper presents a comprehensive overview of the key applications, technical challenges, and future directions of emerging spectroscopic techniques (NIRS, RS, FS, and terahertz spectroscopy), imaging techniques (hyperspectral imaging, MSI, XRI, and thermal imaging), and DL in the context of meat quality and safety inspection. The efficacy of these techniques, often integrated with advanced data analysis methods, has been substantiated through extensive studies on meat quality and safety. These techniques have proven effective in both quantitative and qualitative prediction of quality and safety attributes for the most widely consumed meats globally, including pork, beef, and lamb.

However, the implementation of these emerging technologies also faces challenges related to achieving higher resolution, systematic data processing, and economic considerations. Illumination is a significant factor that limits the accuracy of detection by spectroscopic techniques. This is due to the highly controlled environmental conditions in the laboratory, which may not be the case in industrial environments. The majority of experiments employed for the analysis of meat quality and safety are constrained to laboratory settings due to the expense of the necessary technical equipment for imaging techniques. Nevertheless, the development of advanced processing hardware, software applications, and associated chemometric algorithms, as well as the manufacture of simple yet powerful portable systems, is a key future trend. Specifically, developing portable quality assessment systems that incorporate DL techniques has the potential to positively impact consumer health and ensure competitiveness among producers by reducing image acquisition time, enabling real‐time analysis, and delivering immediate results.

## Author Contributions

**Yushan Jiang:** conceptualization, writing original draft, and investigation. **Dachen Wang:** writing original draft, review and editing. **Zijie Wang:** writing original draft. **Huang Dai:** review and editing. **Chen Wang:** review and editing. **Yingli Wang:** conceptualization, supervision, funding acquisition, and review and editing.

## Funding

This work was supported by the Fundamental Research Funds for the Central Universities (Grant No. 2662023GXQD001).

## Conflicts of Interest

The authors declare no conflicts of interest.

## Data Availability

The data supporting this study's findings are available from the corresponding author upon request.
