# Correction to “Recent Advances in Spectroscopy and Imaging Techniques for Nondestructive Detection of Meat Quality and Safety”

**DOI:** 10.1002/fsn3.71507

**Published:** 2026-02-06

Yushan Jiang, Dachen Wang, Zijie Wang, Huang Dai, Chen Wang, Yingli Wang. Recent Advances in Spectroscopy and Imaging Techniques for Nondestructive Detection of Meat Quality and Safety. *Food Science & Nutrition*. 2025; 12(13): e71303.

In the “Funding” section, the text “This work was supported by the Fundamental Research Funds for the Central Universities (Grant No. 2662023GXQD001)” was incorrect. This should have read: “This work was supported by the Fundamental Research Funds for the Central Universities (Grant No. 2662023GXQD001) and the Hubei Provincial Key Research and Development Program (Grant No: 2024BBB051).”

We apologize for this error.

